# Association between serum vitamin D levels and metabolic dysfunction-associated steatotic liver disease: a cross-sectional study based on NHANES 2021–2023

**DOI:** 10.1097/MS9.0000000000004048

**Published:** 2025-10-14

**Authors:** Shuo Zhou, Xiao You, Dengyong Zhang, Song Yang, Yongliang Chen, Wanliang Sun

**Affiliations:** a School of Medicine, Nankai University, Tianjin, China; b Department of Hepatobiliary Surgery, The First Affiliated Hospital of Bengbu Medical University, Bengbu, China; c The Faculty of Hepatopancreatobiliary Surgery, The First Medical Center, Chinese People’s Liberation Army General Hospital, Beijing, China

**Keywords:** cross-sectional study, MASLD, mediation analysis, NHANES, vitamin D

## Abstract

**Background:**

Metabolic dysfunction-associated steatotic liver disease (MASLD) is a growing global health concern, and emerging evidence suggests a potential role of vitamin D in its pathogenesis. This study aims to evaluate the impact of serum vitamin D levels on the risk of MASLD using National Health and Nutrition Examination Survey (NHANES) data.

**Methods:**

We analyzed data from 3249 US adults based on NHANES 2021–2023 cycle. MASLD was defined by controlled attenuation parameter value ≥248 dB/m. Three multivariable logistic regression models were constructed: Model 1 (vitamin D alone), Model 2 (partially adjusted), and Model 3 (fully adjusted for demographic, metabolic, and inflammatory factors). Model performance was assessed using receiver operating characteristic analysis, calibration curves, and decision curve analysis (DCA). The mediation analysis was used to explore these associations.

**Results:**

The analysis revealed that the MASLD group had significantly lower median vitamin D levels (77.35 vs. 82.10 nmol/L, *P* < 0.001) and a higher percentage in the lowest vitamin D quartile (27.2% vs. 22.1%, *P* < 0.001). The multivariable logistic regression demonstrated an inverse association between vitamin D and MASLD prevalence, with each 10 nmol/L increase correlating with a 3.5% reduction in MASLD risk [odds ratio (OR) 0.965, 95% confidence interval (CI) 0.941, 0.989, *P* = 0.005]. Restricted cubic splines confirmed a linear dose-response relationship (*P* nonlinear = 0.915). The fully adjusted model 3 showed excellent discrimination (AUC 0.825, 95% CI 0.811, 0.839), indicating excellent predictive performance for MASLD risk assessment. DCA further established that the fully adjusted model provided clinically meaningful net benefits across a wide threshold probability range (15–90%) and prevented 38–42% of unnecessary interventions for MASLD management. Mediation analysis indicated that body mass index, high-density lipoprotein cholesterol, and glycohemoglobin mediated 52.11, 19.12, and 6.95% of the association of vitamin D and MASLD.

**Conclusions:**

Our findings suggest that higher vitamin D levels were associated with lower odds of MASLD, highlighting its potential as a modifiable risk factor and a promising therapeutic target.

## Introduction

Metabolic dysfunction-associated steatotic liver disease (MASLD) has emerged as a significant public health concern, characterized by excessive fat accumulation in the liver without alcohol consumption. This condition is increasingly recognized as the hepatic manifestation of metabolic syndrome, with its prevalence rising alongside the global obesity epidemic^[1,2]^. MASLD encompasses a spectrum of liver disorders, ranging from simple steatosis to more severe forms such as Metabolic dysfunction-associated steatohepatitis (MASH), which can progress to cirrhosis and even hepatocellular carcinoma, representing a major cause of morbidity and mortality worldwide^[3,4]^. The economic burden of MASLD is substantial, placing considerable strain on health care systems^[5]^.

**HIGHLIGHTS**

- This study, based on National Health and Nutrition Examination Survey 2021–2023 data, is the first to integrate mediation analysis and advanced predictive modeling (receiver operating characteristic and decision curve analysis) to clarify the role of vitamin D in Metabolic dysfunction-associated steatotic liver disease (MASLD), highlighting its dose-dependent protective effect.
- This study revealed a significant inverse association between serum vitamin D levels and MASLD prevalence. The fully adjusted model demonstrated excellent predictive performance for MASLD risk stratification. The mediation analysis were also used to explore these associations.
- Vitamin D status may serve as a modifiable risk factor for MASLD prevention, particularly in high-risk groups. Clinical strategies should consider combined interventions targeting both vitamin D supplementation and metabolic mediators. Prospective studies are needed to establish causality and evaluate vitamin D supplementation’s efficacy in MASLD management.

Currently, the diagnostic approaches for MASLD primarily rely on imaging techniques and liver biopsy, although it is the gold standard, but which are invasive and carry inherent risks^[6]^. The non-invasive methods, including serum biomarker assessments, are under investigation to facilitate early diagnosis and monitor disease progression^[7]^. Treatment options for MASLD remain limited, with lifestyle modifications, such as weight loss and dietary changes, being the primary recommendation. Pharmacological interventions are still largely experimental, underscoring the need for innovative therapeutic strategies^[8,9]^.

Vitamin D has been shown to exhibit anti-inflammatory, anti-fibrotic, and immunomodulatory effects. A deficiency in this vitamin, indicated by low serum levels of 25-hydroxyvitamin D [25(OH)D], is associated with increased risks of cardiovascular disease, metabolic syndrome, and type 2 diabetes. The relationship between vitamin D deficiency and MASLD remains controversial. Evidence suggests that low vitamin D levels may be associated with increased liver fat accumulation and inflammation, implicating the vitamin in metabolic processes relevant to liver health^[10–12]^. In contrast, another study found no association between vitamin D concentrations and MASLD^[13]^. Further supporting this discrepancy, a recent bidirectional Mendelian randomization analysis revealed no causal relationship between vitamin D status and ultrasound-diagnosed non-alcoholic fatty liver disease^[14]^. Recently, Resmetirom (Rezdiffra) is an oral thyroid hormone receptor-β agonist that was approved for use in conjunction with diet and exercise for the treatment of adults with noncirrhotic MASH with moderate to advanced liver fibrosis in the USA^[15]^. However, the underlying mechanisms through which vitamin D influences liver pathology remain poorly understood, necessitating further exploration into its role in MASLD pathogenesis^[16]^.

Our study employs a cross-sectional design utilizing data from the National Health and Nutrition Examination Survey (NHANES), providing a representative sample of the US population. The NHANES dataset allows for robust statistical analyses, enabling thorough covariate adjustments to ascertain the relationship between serum vitamin D levels and MASLD prevalence. The findings may also offer valuable insights for developing targeted clinical interventions and public health strategies to mitigate the burden of MASLD, with the ultimate goal of informing evidence-based prevention approaches.

## Materials and methods

### Study population

This cross-sectional study analyzed data from the 2021–2023 NHANES, a nationally representative health assessment of the US population. The initial sample included 11 933 participants, from which we excluded individuals under 20 years old, those with missing data, patients with chronic hepatitis B, and long-term alcohol users. The final analytical cohort consisted of 3249 adults who had complete liver transient elastography results and serum vitamin D measurements. All study procedures followed the NHANES protocol approved by the National Center for Health Statistics Ethics Review Board, ensuring compliance with ethical research standards. This selection process yielded a representative sample for investigating the association between vitamin D status and liver health in American adults.

### Measurement of MASLD and serum vitamin D

The transient elastography measurements were obtained in the NHANES Mobile Examination Center, using the FibroScan model 502 V2 Touch equipped with a medium or extra-large wand (probe). Liver steatosis and fibrosis were evaluated using FibroScan, which combines ultrasound with vibration-controlled (low frequency 50 Hz) elastography to measure liver stiffness and the controlled attenuation parameter (CAP, dB/m) for hepatic fat quantification. A minimum of 10 valid measurements were obtained per participant, with median CAP and liver stiffness measurement (LSM, kPa) values automatically calculated alongside interquartile ranges (IQR). Quality control required an IQR/M ratio <30%. MASLD was defined as a CAP ≥ 248 dB/m^[17]^, a validated threshold for hepatic steatosis. Standardized protocols ensured measurement accuracy, with trained technicians performing all assessments. The total 25(OH)D levels in serum was calculated as the sum of 25(OH)D2 and 25(OH)D3, which were quantified using high-performance liquid chromatography-tandem mass spectrometry at the National Center for Environmental Health. Participants were categorized into vitamin D quartiles based on serum concentrations: Q1 (≤58.10 nmol/L), Q2 (58.20–78.80 nmol/L), Q3 (78.90–102.00 nmol/L), and Q4 (≥103.00 nmol/L). The lowest quartile (Q1) served as the reference group for comparative analyses.

### Covariates

This study analyzed baseline data from 3249 NHANES participants, collecting self-reported demographic and clinical characteristics including gender (male, female), age (years), race (Mexican American, Non-Hispanic White, Non-Hispanic Black, Other Hispanic, and Other Race), education level (≤high school or >high school), diabetes status (yes, no), hypertension (yes, no), smoking (yes, no), and kidney dysfunction (yes, no), along with physical examination measures of body mass index (BMI) categorized as underweight/normal (<25 kg/m^2^), overweight (25–30 kg/m^2^), or obese (≥30 kg/m^2^), and laboratory assessments of serum biomarkers comprising total cholesterol (mmol/L), high-density lipoprotein cholesterol (HDL-C, mmol/L), high-sensitivity C-reactive protein (CRP, mg/L), glycohemoglobin (%), and complete blood count parameters (White Blood Cell (WBC), neutrophils, lymphocytes, and monocytes, ×10^9^/L). All laboratory indicators were obtained by measuring the serum samples.

### Statistical analysis

All statistical analyses were conducted using R (version 4.3.0) and SPSS (version 26.0), with significance defined as two-tailed *P* < 0.05. Categorical data were expressed as frequencies (%) and analyzed by *χ*^2^/Fisher’s exact tests, while non-normal continuous variables were presented as medians (IQR) and compared using Mann–Whitney *U* tests. Accounting for NHANES’s complex sampling, weighted analyses ensured national representativeness. We developed three predictive models through weighted multivariable logistic regression, analyzing vitamin D both as continuously and categorical(quartile) variables, with adjustment for relevant MASLD covariates. Our comprehensive analytical framework incorporated restricted cubic splines (RCS) to evaluate potential dose-response relationships, receiver operating characteristic (ROC) analysis with C-index calculation to assess model discrimination, and decision curve analysis (DCA) to examine the clinical utility of all three prediction models. Additional subgroup analyses were performed to investigate population-specific vitamin D-MASLD associations, collectively providing a comprehensive evaluation of both statistical significance and clinical relevance. The “mediation” package in R 4.3.0. was utilized to perform Mediation analysis assessing the mediating effects of metabolism indicators (BMI, HDL-C, and glycohemoglobin) on the associations of vitamin D (continuous variable) and MASLD (measured by CAP value), adjusted by age, gender, race, education-level, diabetes, hypertension, smoking, kidneys-weak, BMI, WBC, neutrophils, lymphocyte, monocyte, CRP, and glycohemoglobin. The presence of a mediating effect was defined as satisfying all of the following conditions having a significant indirect effect, a significant total effect, and a positive proportion of the mediator effect.

## Results

### Demographical and clinical characteristics of the study population

**Figure 1.**
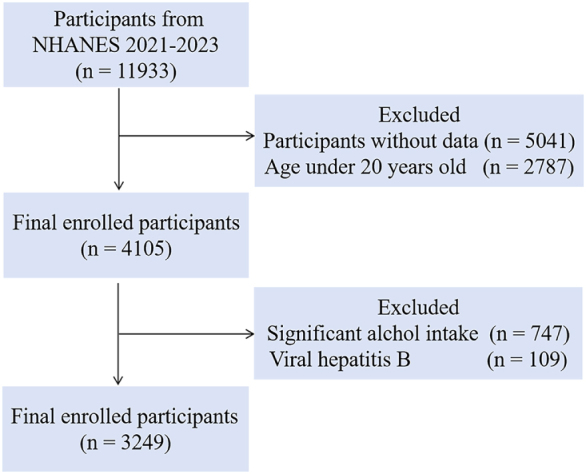
Flowchart showing the selection of study population.

The study population comprised 3249 participants, including 1427 non-MASLD and 1822 MASLD cases (Fig. 1). The baseline characteristics of all participants were showed in Table 1 and Table 2. There is a significant differences between MASLD and non-MASLD groups across multiple variables. Demographically, MASLD prevalence was associated with male (46.82% vs. 40.85%, *P* < 0.001), lower education level (≤high school: 29.64% vs. 21.44%, *P* < 0.001), higher rates of diabetes (19.70% vs. 7.29%, *P* < 0.001) and hypertension (42.92% vs. 23.90%, *P* < 0.001). Metabolic parameters showed marked deterioration in MASLD, including patients were significantly older (median 59 vs. 52 years, *P* <0.001), with elevated BMI (31.1 vs. 25.2 kg/m^2^, *P* < 0.001), dyslipidemia (HDL-C 1.29 vs. 1.50 mmol/L, *P* < 0.001), and poorer glycemic control (glycohemoglobin 5.6% vs. 5.4%, *P* < 0.001). The MASLD group demonstrated significantly higher inflammatory markers (CRP 2.53 vs. 1.08 mg/L; WBC 6.9 vs. 6.1 × 10^9^/L) and LSM (5.5 vs 4.5 kPa, *P* < 0.001). Notably, vitamin D deficiency was more prevalent in MASLD, with lower median levels (77.35 vs. 82.10 nmol/L) and overrepresentation in the lowest quartile (27.2% vs. 22.1%, *P* < 0.001). There was no significant difference in the proportion of kidneys weak between the two groups (*P* > 0.05). Our findings comprehensively demonstrate the multisystemic nature of MASLD, encompassing metabolic, inflammatory, and sociodemographic dimensions.

**Table 1:** Baseline characteristics of the NHANES 2021–2023 study population

Variables	Total	Non-MASLD	MASLD	*P*
	*N* = 3249	*N* = 1427	*N* = 1822	
Age (years), *n* (%)				<0.001
<60	1771 (54.00, 51.00)	851 (59.00, 64.00)	920 (50.00, 49.00)	
≥60	1478 (45.00, 49.00)	576 (40.00, 36.00)	902 (49.00, 51.00)	
Gender, *n* (%)				<0.001
Female	1813 (55.80)	844 (59.15)	969 (53.18)	
Male	1436 (44.20)	583 (40.85)	853 (46.82)	
Education level, *n* (%)				<0.001
>High school	2403 (73.96)	1121 (78.56)	1282 (70.36)	
≤High school	846 (26.04)	306 (21.44)	540 (29.64)	
Race, *n* (%)				0.007
Mexican American	208 (6.40)	72 (5.05)	136 (7.46)	
Non-Hispanic Black	338 (10.40)	172 (12.05)	166 (9.11)	
Non-Hispanic White	2087 (64.24)	914 (64.05)	1173 (64.38)	
Other Hispanic	289 (8.90)	124 (8.69)	165 (9.06)	
Other Race	327 (10.06)	145 (10.16)	182 (9.99)	
Smoking, *n* (%)				<0.001
No	2090 (64.33)	973 (68.19)	1117 (61.31)	
Yes	1159 (35.67)	454 (31.81)	705 (38.69)	
Diabetes, *n* (%)				<0.001
No	2786 (85.75)	1323 (92.71)	1463 (80.30)	
Yes	463 (14.25)	104 (7.29)	359 (19.70)	
Hypertension, *n* (%)				<0.001
No	2126 (65.44)	1086 (76.10)	1040 (57.08)	
Yes	1123 (34.56)	341 (23.90)	782 (42.92)	
Kidneys weak, *n* (%)				0.628
No	3162 (97.32)	1391 (97.48)	1771 (97.20)	
Yes	87 (2.68)	36 (2.52)	51 (2.80)	
Vitamin D (nmol/L), *n* (%)				<0.001
Q1 (≤58.10)	811 (24.96)	316 (22.14)	495 (27.17)	
Q2 (58.20–78.80)	811 (24.96)	346 (24.25)	465 (25.52)	
Q3 (78.90–102.00)	800 (24.62)	359 (25.16)	441 (24.20)	
Q4 (≥103.00)	827 (25.45)	406 (28.45)	421 (23.11)	

Data are presented as median (interquartile range), or number (%) of participants with a condition.

MASLD, metabolic dysfunction-associated steatotic liver disease.

**Table 2:** Demographical characteristics of the NHANES 2021–2023 study population

Variables	Total	Non-MASLD	MASLD	*P*
	*N* = 3249	*N* = 1427	*N* = 1822	
Age (years)	57 (39.00, 67.00)	52.00 (34.50, 67.00)	59.00 (44.00, 68.00)	<0.001
BMI (kg/m^2^)	28.40 (24.70, 33.30)	25.20 (22.70, 28.30)	31.10 (27.60, 35.90)	<0.001
Vitamin D (nmol/L)	78.90 (58.20, 103.00)	82.10 (61.60, 106.00)	77.35 (55.60, 99.20)	<0.001
LSM (kPa)	5.00 (4.10, 6.30)	4.50 (3.70, 5.50)	5.50 (4.40, 7.00)	<0.001
Total cholesterol (mmol/L)	4.84 (4.16, 5.56)	4.76 (4.14, 5.46)	4.90 (4.19, 5.64)	0.001
HDL-C (mmol/L)	1.40 (1.16, 1.63)	1.50 (1.27, 1.76)	1.29 (1.09, 1.53)	<0.001
Glycohemoglobin (%)	5.50 (5.20, 5.80)	5.40 (5.10, 5.60)	5.60 (5.30, 6.00)	<0.001
CRP (mg/L)	1.72 (0.79, 3.92)	1.08 (0.57, 2.40)	2.53 (1.16, 5.08)	<0.001
WBC (10^9^/L)	6.60 (5.40, 7.80)	6.10 (5.10, 7.30)	6.90 (5.80, 8.20)	<0.001
Lymphocyte (10^9^/L)	1.90 (1.50, 2.40)	1.80 (1.50, 2.20)	2.00 (1.60, 2.50)	<0.001
Neutrophils (10^9^/L)	3.80 (3.00, 4.80)	3.50 (2.70, 4.50)	4.00 (3.20, 5.10)	<0.001
Monocyte (10^9^/L)	0.50 (0.40, 0.60)	0.50 (0.40, 0.60)	0.50 (0.40, 0.60)	<0.001

Data are presented as median (interquartile range) of participants with a condition.

BMI, body mass index; CRP, C-reactive protein; HDL-C, high-density lipoprotein cholesterol; LSM, liver stiffness measurements; MASLD, metabolic dysfunction-associated steatotic liver disease.

### Associations between Vitamin D and prevalence of MASLD

Multi-variable analyses demonstrated a significant inverse association between serum vitamin D levels and MASLD prevalence across all models (Table 3). In the fully adjusted model (Model 3), each 10 nmol/L increase in vitamin D concentration was associated with a 3.5% reduction in MASLD risk (OR = 0.965, 95% CI 0.941, 0.989, *P* = 0.005). When analyzed by quartiles, participants in the highest vitamin D quartile (Q4) showed a 29.5% lower MASLD risk compared to the lowest quartile (Q1; OR 0.705, 95% CI 0.540, 0.921, *P* = 0.01), with a significant dose-response trend (*P* for trend <0.001). The protective association was most pronounced in Model 2, where Q4 participants had a 52.4% risk reduction (OR 0.476, 95% CI 0.379, 0.598, *P* < 0.001). While the middle quartiles (Q2 and Q3) showed non-significant associations in the fully adjusted model, the consistent inverse trends across all models and significant *P*-values for trend tests support a potential protective role of higher vitamin D levels against MASLD development.

**Table 3:** Weighted multi-variate logistic regression of the association between vitamin D and MASLD

Vitamin D (10 nmol/L)	Model 1 OR (95% CI)	*P*	Model 2 OR (95% CI)	*P*	Model 3 OR (95% CI)	*P*
Continuous variable	0.962 (0.945, 0.980)	<0.001	0.933 (0.912, 0.953)	<0.001	0.965 (0.941, 0.989)	0.005
Quartile variable						
Q1	Reference		Reference		Reference	
Q2	0.858 (0.704, 1.046)	0.130	0.784 (0.635, 0.967)	0.023	0.875 (0.683, 1.121)	0.291
Q3	0.784 (0.643, 0.956)	0.016	0.664 (0.534, 0.827)	<0.001	0.612 (0.725, 1.211)	0.618
Q4	0.662 (0.544, 0.805)	<0.001	0.476 (0.379, 0.598)	<0.001	0.705 (0.540, 0.921)	0.010
P for trend		<0.001		0.044		<0.001

CI, confidence interval; OR, odds ratio.

Model 1: unadjusted.

Model 2: Adjust: age, gender, race, education level, diabetes, hypertension, and smoking.

Model 3: Adjust: age, gender, race, education level, diabetes, hypertension, smoking, BMI, WBC, lymphocyte, total cholesterol, HDL-C, CRP, and glycohemoglobin.

### Subgroup analysis on the associations between vitamin D and MASLD

**Figure 2.**
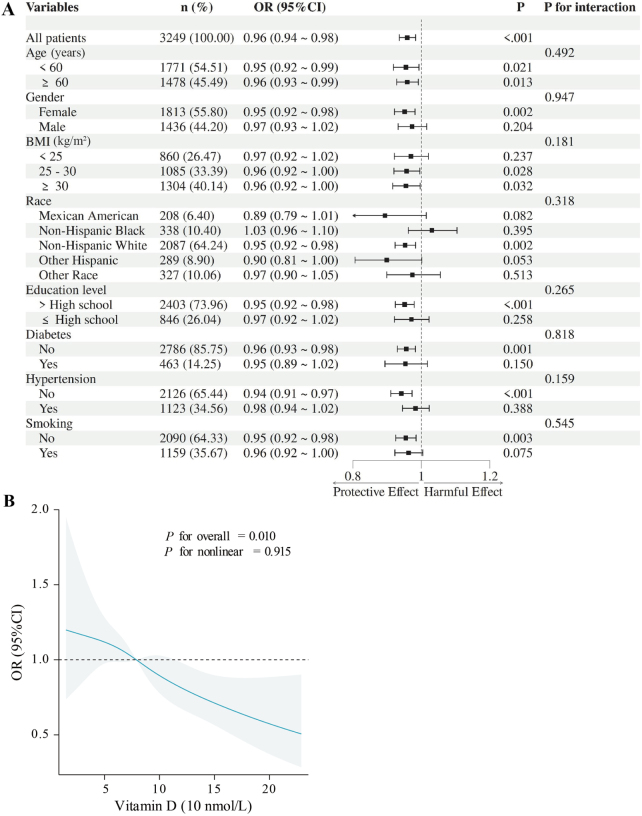
Association between vitamin D and prevalence of MASLD. (A) Subgroup analysis of clinical characteristics. (B) RCS curve of the model. Values were adjusted for age, gender, race, education level, diabetes, hypertension, smoking, BMI, WBC, lymphocyte, total cholesterol, HDL-C, CRP, and glycohemoglobin. BMI, body mass index; HDL-C, high-density lipoprotein cholesterol; CRP, high-sensitivity C-reactive protein; RCS, restricted cubic spline; OR, odds ratio.

Subgroup analyses demonstrated a consistent inverse association between serum vitamin D levels (per 10 nmol/L increase) and MASLD prevalence in the overall population (OR 0.96, *P* < 0.001; Fig. 2A). The protective effect remained significant across most subgroups including both age categories (<60 years: OR 0.95; ≥60 years: OR 0.96), females (OR 0.95), normal weight to obese individuals (BMI 25–30: OR 0.96; BMI ≥ 30: OR 0.96), and non-Hispanic Whites (OR 0.95; all *P* < 0.05). Notably stronger associations were observed in non-hypertensive participants (OR 0.94, *P* < 0.001) and those with higher education levels (OR 0.95, *P* < 0.001). No significant interactions were found by all subgroups above in the Fig. 2A (all *P* for interaction >0.05), indicating the vitamin D and MASLD relationship was largely independent of these factors. While some subgroups (e.g., men and Mexican Americans) showed an insignificant trend, the overall homogeneity of the effect in the population (*P* < 0.001 for all patients) suggests that the protective effect of vitamin D may be broadly universal. The RCS analysis model with four knots(placed at the 5th, 35th, 65th, and 95th percentiles) revealed a significant overall relationship between serum vitamin D levels and MASLD risk (*P* for overall = 0.01; Fig. 2B), indicating that vitamin D is an important predictor of MASLD. However, the *P*-value for nonlinearity was 0.915, suggesting that the relationship between vitamin D (per 10 nmol/L increase) and MASLD follows a linear pattern rather than a nonlinear or U-shaped curve, which also supporting the use of a linear model in subsequent analyses.

### Predictive value of vitamin D on the prevalence of MASLD

**Figure 3.**
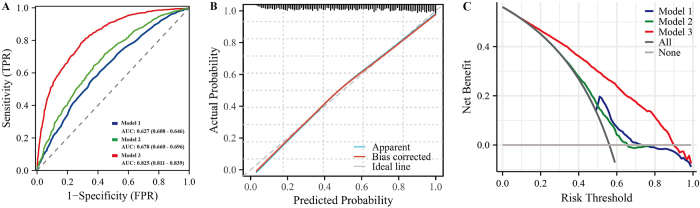
Predictive curve of vitamin D and prevalence of MASLD. (A) ROC curve of the model. (B) Calibration curve of the model. (C) DCA curve of the model. Model 1: Unadjusted. Model 2: Adjust: age, gender, race, education level, diabetes, hypertension, and smoking. Model 3: Adjust: age, gender, race, education level, diabetes, hypertension, smoking, BMI, WBC, lymphocyte, total cholesterol, HDL-C, CRP, and glycohemoglobin. ROC, receiver operating characteristic; DCA, decision curve analysis; BMI, body mass index; HDL-C, high-density lipoprotein cholesterol; CRP, high-sensitivity C-reactive protein.

The ROC curve analysis revealed a graded improvement in MASLD prediction accuracy across the three models (Fig. 3A), with Model 3 (Area Under the Curve (AUC) = 0.825, 95% CI 0.811, 0.839) demonstrating significantly superior discriminative performance compared to both Model 2 (AUC = 0.678, 95% CI 0.660, 0.696; DeLong’s test < 0.001) and Model 1 (AUC = 0.627, 95% CI 0.608, 0.646; DeLong’s test *P* < 0.001). The model demonstrated excellent discrimination for MASLD risk prediction, with a C-index of 0.832 (95% CI 0.819, 0.845). Calibration analysis revealed good agreement between predicted and observed probabilities (Hosmer-Lemeshow goodness-of-fit test: *P* = 0.093), indicating no significant deviation from perfect calibration. The likelihood ratio test was highly significant (*P* < 0.001), confirming the overall model fit. Multicollinearity assessment showed that all variance inflation factors were below 5 (Supplemental Digital Content Appendix Table S1, available at http://links.lww.com/MS9/A983), suggesting no concerning collinearity among predictors (Fig. 3B). DCA demonstrated progressively wider clinical utility across the three models in this MASLD cohort (Fig. 3C). Model 1 provided net benefit within a limited threshold probability range of 48–70%, while Model 2 extended this range to 40–65%. The fully adjusted Model 3 showed superior performance, offering clinically meaningful net benefit across nearly the entire decision spectrum (15–90% thresholds). All models showed greater net benefit than the “treat-all” or “treat-none” strategies.

### Mediating effect on the association between Vitamin D and MASLD

**Figure 4.**
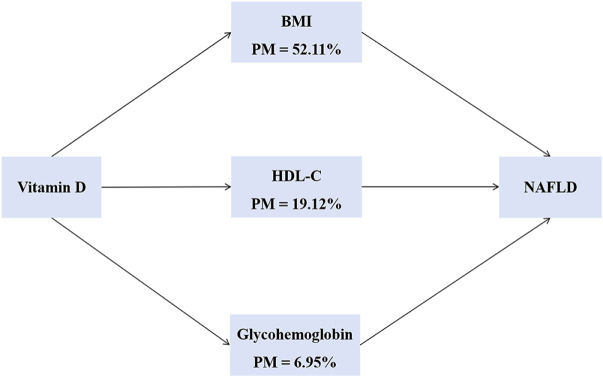
Mediating role of metabolism related indicators. PM, proportion of mediation. Adjust: age, gender, race, education-level, diabetes, hypertension, smoking, kidneys_weak, BMI, WBC, neutrophils, lymphocyte, monocyte, CRP, and glycohemoglobin.

The mediation analysis revealed significant indirect correlation of serum vitamin D levels on MASLD (measured by CAP value) through BMI, HDL-C, and glycohemoglobin metabolic pathways (Fig. 4). As shown in Supplemental Digital Content Appendix Table S2, available at http://links.lww.com/MS9/A984. BMI demonstrated the strongest mediating effect (*β* = −0.09), accounting for 52.11% of the total effect. HDL-C showed a modest but significant mediation [*β* = −0.02, proportion of mediation (PM) = 19.12%], supporting vitamin D’s role in improving lipid metabolism. In contrast, glycohemoglobin exhibited minimal mediation (*β* = −0.01, PM = 6.95%), suggesting that vitamin D’s benefits on MASLD are largely independent of glycemic control. Notably, vitamin D retained a significant direct correlation on MASLD (*β* = −0.08, *P* < 0.001) after accounting for all mediators.

## Discussion

MASLD has become a major global health challenge, representing a spectrum of hepatic disorders that progress from simple steatosis to MASH, with potential advancement to cirrhosis and hepatocellular carcinoma^[1]^. As the predominant cause of chronic liver disease, the disease’s escalating prevalence reflects the global epidemic of metabolic disorders, understanding its pathophysiology, risk factors, and potential preventive strategies is critical for reducing the burden of this disease on health care systems^[18–20]^. Previous research has suggested that low levels of vitamin D may be associated with an increased risk of developing MASLD, but it remains controversial^[21,22]^. This cross-sectional study investigates the deeper relationship between serum vitamin D levels and liver health based on NHANES 2021–2023 cycle, specifically focusing on the prevalence of MASLD. We constructed a comprehensive model combining vitamin D with metabolic factors that provides superior risk prediction and clinical utility across a wide range of intervention thresholds, indicating the potential role of vitamin D in MASLD prevention and liver metabolism. The findings from this research could provide insights into vitamin D status as a modifiable risk factor for MASLD, contributing to the development of targeted preventive measures.

The innovation of this study lies in its examination of the relationship between serum vitamin D levels and MASLD in a large, representative population sample. Our findings provide novel insights into the potential protective role of vitamin D against MASLD in humans, unlike previous research that primarily focused on animal models or small cohorts^[23,24]^. According to the forest plot results, the strongest protective effect was seen in metabolically healthy subgroups (non-diabetic and non-hypertensive), and the protective effect was weakened in high-risk groups, but there was still a trend. The RCS curve results support a consistent, dose-dependent decrease in MASLD risk with higher vitamin D levels, without evidence of a threshold effect or increased risk at either extreme of vitamin D concentration. While vitamin D alone showed modest predictive capacity (AUC = 0.627), its combination with additional risk factors in Model 2 yielded intermediate performance (AUC = 0.678), and the fully adjusted Model 3 achieved excellent diagnostic accuracy (AUC = 0.825). These results demonstrate that vitamin D serves as an important but insufficient standalone predictor, and its clinical utility for MASLD screening is markedly enhanced when integrated into a multifactorial risk assessment model. Our research data supported a linear association. However, in Model 3, the Q2 and Q3 groups showed non-significant *P*-values, while the Q4 group reached statistical significance. This pattern implies that the relationship between vitamin D and MASLD may exhibit a threshold effect rather than a simple dose-response relationship. Specifically, vitamin D may need to reach a certain threshold level (e.g., Q4 concentration) to demonstrate protective effects against MASLD. Further large-scale studies are still warranted to confirm this potential dose-response pattern. Previous studies have established correlations between vitamin D deficiency and liver disease^[10,16,22]^, yet our research demonstrate this association using comprehensive data from the NHANES on a large scale. This establishes a critical link in understanding the pathophysiology of MASLD and suggests that vitamin D may serve as a modifiable risk factor in its prevention and management.

These results collectively validate the model’s robustness in both discriminating MASLD cases and accurately estimating risk probabilities across the spectrum of vitamin D levels^[25]^. This expansion of useful threshold ranges – from Model 1’s intermediate-risk focus to Model 3’s coverage of both conservative (15%) and aggressive (90%) intervention preferences – indicates that comprehensive risk factor integration substantially enhances clinical applicability for MASLD management decisions. The results suggest that Model 3 could guide interventions across diverse clinical scenarios while avoiding 38–42% of unnecessary treatments in low-risk groups (15–30% thresholds). These results demonstrate that the comprehensive Model 3 offers reliable clinical utility across the entire spectrum of decision thresholds for MASLD risk assessment, which superior to both simpler models and current guideline-based approaches while maintaining robust statistical reliability^[26]^. In our study, we further explored the potential mediating effect of metabolism related indicators within these associations. Our results indicate that vitamin D may mitigate adiposity through regulating adipocyte differentiation and inflammatory responses^[27,28]^, consequently decreasing hepatic lipid accumulation^[29]^. Combined interventions of vitamin D supplementation and weight management could yield synergistic effects in MASLD prevention. Additionally, vitamin D appears to improve HDL functionality by enhancing reverse cholesterol transport, thereby reducing hepatic lipid deposition^[30,31]^. This suggests that strategies to elevate HDL-C levels may augment vitamin D’s protective effects. The minimal mediation through glycohemoglobin implies vitamin D’s MASLD benefits are largely independent of glycemic control in this cohort, indicating limited efficacy of vitamin D monotherapy for glycemic-related MASLD prevention^[32]^. Notably, vitamin D retained a significant direct effect on MASLD (*β* = −0.08, *P* < 0.001) after accounting for all mediators, implying additional protective mechanisms beyond these metabolic pathways^[33–35]^. Our findings highlight BMI as the primary mediator and suggest that combined strategies targeting both vitamin D status and metabolic factors may be most effective for MASLD prevention. The implications of our findings extend into clinical practice and public health policy. Given the significant inverse relationship between vitamin D levels and MASLD prevalence, there is a compelling case for integrating vitamin D status assessments into routine clinical evaluations, especially for high-risk populations such as older adults and those with obesity-related comorbidities. Based on our quartile analysis, we now suggest a target vitamin D level of >80 nmol/L and recommend prioritizing trials in obese and hypertensive subgroups. The potential for vitamin D supplementation as a preventive strategy against liver disease could significantly alter the management landscape for MASLD, which currently lacks effective pharmacological treatments^[36,37]^. As public health initiatives increasingly focus on lifestyle modifications and prevention strategies, this research underscores the importance of vitamin D not only as a nutrient but as a crucial component of liver health management^[38,39]^.

However, the limitations of this cross-sectional research study warrant careful consideration. First, the inherent selection bias associated with cross-sectional designs may restrict the generalizability of the findings, as the NHANES cohort may not represent the broader population adequately. Second, the study’s reliance on self-reported data introduces the potential for inaccuracies, particularly regarding lifestyle factors (e.g., dietary intake, physical activity, and sun exposure) and comorbidities. Lastly, the absence of longitudinal and interventional studies restricts our ability to infer causality, leaving the temporal relationship between vitamin D levels and MASLD prevalence ambiguous to some extent. These limitations emphasize the need for further investigations employing longitudinal designs and objective measures to validate the observed associations and enhance our understanding of the role of vitamin D in MASLD progression and treatment.

## Conclusions

In conclusion, this study elucidates a significant inverse relationship between serum vitamin D levels and the prevalence of MASLD, suggesting that higher vitamin D status was associated with lower odds of MASLD. These findings emphasize the potential for vitamin D as a modifiable risk factor in MASLD management and highlight the necessity for future research to explore targeted interventions aimed at improving vitamin D levels among at-risk populations. By advancing our understanding of vitamin D in liver health, we can potentially contribute to the development of effective preventive strategies against MASLD and its associated complications.

## Data Availability

The complete data about NHANES details are publicly available on the internet for researchers and can be accessed from (https://www.cdc.gov/nchs/nhanes/). Data will be made available by the authors upon reasonable request.

## Research registration unique identifying number (UIN)

This research studies involving human participants had being registered in a publicly accessible NHANES database. The complete data about NHANES details are publicly available on the internet for researchers and can be accessed from https://www.cdc.gov/nchs/nhanes/.

## References

[1] Eslam M Sanyal AJ George J. International consensus panel. MAFLD: a consensus-driven proposed nomenclature for metabolic associated fatty liver disease. Gastroenterology 2020;158:1999–2014. 32044314 10.1053/j.gastro.2019.11.312

[2] Rinella ME Lazarus JV Ratziu V. A multisociety Delphi consensus statement on new fatty liver disease nomenclature. J Hepatol 2023;79:1542–56. 37364790 10.1016/j.jhep.2023.06.003

[3] Hagström H Shang Y Hegmar H. Natural history and progression of metabolic dysfunction-associated steatotic liver disease. Lancet Gastroenterol Hepatol 2024;9:944–56. 39243773 10.1016/S2468-1253(24)00193-6

[4] Golabi P Owrangi S Younossi ZM. Global perspective on nonalcoholic fatty liver disease and nonalcoholic steatohepatitis-prevalence, clinical impact, economic implications and management strategies. Aliment Pharmacol Ther 2024;18:884–96. 10.1111/apt.17833 38813821

[5] Zhang JJ Yu HC Li Y. Association between serum 25-hydroxy vitamin D concentrations and mortality among individuals with metabolic dysfunction-associated fatty liver disease: a prospective cohort study. Am J Clin Nutr 2022;116:1409–17. 36107812 10.1093/ajcn/nqac260

[6] Wan Q Liu X Xu J. Body composition and progression of biopsy-proven non-alcoholic fatty liver disease in patients with obesity. J Cachexia Sarcopenia Muscle 2024;15:2608–17. 39389917 10.1002/jcsm.13605 PMC11634503

[7] Sanyal AJ Castera L Wong VW. Noninvasive Assessment of Liver Fibrosis in NAFLD. Clin Gastroenterol Hepatol 2023;21:2026–39. 37062495 10.1016/j.cgh.2023.03.042

[8] Qin S Cheng X Zhang S. Sleep patterns, genetic susceptibility, and risk of cirrhosis among individuals with nonalcoholic fatty liver disease. Hepatol Int 2024;18:1158–67. 38888882 10.1007/s12072-024-10665-7

[9] Singal AK Shah VH Malhi H. Emerging targets for therapy in ALD: lessons from NASH. Hepatology 2024;80:223–37. 36938877 10.1097/HEP.0000000000000381 PMC10511666

[10] Ji Y Wei CB Gu W. Relevance of vitamin D on NAFLD and liver fibrosis detected by vibration controlled transient elastography in US adults: a cross-sectional analysis of NHANES 2017-2018. Ann Med 2023;55:2209335. 37155562 10.1080/07853890.2023.2209335 PMC10167885

[11] Chung SI Liang L Han H. Vitamin D attenuates non-alcoholic fatty liver disease in high-fat diet-induced obesity murine model. Yonsei Med J 2025;66:75–86. 39894040 10.3349/ymj.2024.0038 PMC11790407

[12] Fang A Zhao Y Yang P. Vitamin D and human health: evidence from Mendelian randomization studies. Eur J Epidemiol 2024;39:467–90. 38214845 10.1007/s10654-023-01075-4

[13] Lee HK Shin SR Han AL. Metabolic dysfunction associated fatty liver disease (MAFLD) and serum vitamin D concentration in South Korea. Asia Pac J Clin Nutr 2022;31:201–07. 35766555 10.6133/apjcn.202206_31(2).0005

[14] Wang N Chen C Zhao L. Vitamin D and nonalcoholic fatty liver disease: bi-directional mendelian randomization analysis. EBioMedicine 2018;28:187–93. 29339098 10.1016/j.ebiom.2017.12.027 PMC5835542

[15] Keam SJ. Resmetirom: first approval. Drugs 2024;84:729–35. 38771485 10.1007/s40265-024-02045-0

[16] Keane JT Elangovan H Stokes RA. Vitamin D and the Liver-Correlation or Cause? Nutrients 2018;10:496. 29659559 10.3390/nu10040496 PMC5946281

[17] Karlas T Petroff D Sasso M. Individual patient data meta-analysis of controlled attenuation parameter (CAP) technology for assessing steatosis. J Hepatol 2017;66:1022–30. 28039099 10.1016/j.jhep.2016.12.022

[18] Vaz K Kemp W Majeed A. NAFLD and MAFLD independently increase the risk of major adverse cardiovascular events (MACE): a 20-year longitudinal follow-up study from regional Australia. Hepatol Int 2024;18:1135–43. 39008030 10.1007/s12072-024-10706-1 PMC11297804

[19] Zheng H Sechi LA Navarese EP. Metabolic dysfunction-associated steatotic liver disease and cardiovascular risk: a comprehensive review. Cardiovasc Diabetol 2024;23:346. 39342178 10.1186/s12933-024-02434-5 PMC11439309

[20] Gawrieh S Vilar-Gomez E Wilson LA. NASH clinical research network. Increases and decreases in liver stiffness measurement are independently associated with the risk of liver-related events in NAFLD. J Hepatol 2024;81:600–08. 38762169 10.1016/j.jhep.2024.05.008 PMC11410523

[21] Wang Q Shi X Wang J. Low serum vitamin D concentrations are associated with obese but not lean NAFLD: a cross-sectional study. Nutr J 2021;20:30. 33794916 10.1186/s12937-021-00690-9 PMC8017627

[22] Yuan S Larsson SC. Inverse association between serum 25-Hydroxyvitamin D and nonalcoholic fatty liver disease. Clin Gastroenterol Hepatol 2023;21:398–405. 35101633 10.1016/j.cgh.2022.01.021

[23] Dong B Zhou Y Wang W. Vitamin D receptor activation in liver macrophages ameliorates hepatic inflammation, steatosis, and insulin resistance in mice. Hepatology 2020;71:1559–74. 31506976 10.1002/hep.30937

[24] Yang A Chen Y Gao Y. Vitamin D3 exacerbates steatosis while calcipotriol inhibits inflammation in non-alcoholic fatty liver disease in Sod1 knockout mice: a comparative study of two forms of vitamin D. Food Funct 2024;15:4614–26. 38590249 10.1039/d4fo00215f

[25] Zhang Z Moon R Thorne JL. NAFLD and vitamin D: evidence for intersection of microRNA-regulated pathways. Nutr Res Rev 2023;36:120–39. 35109946 10.1017/S095442242100038X

[26] European Association for the Study of the Liver (EASL). European Association for the Study of Diabetes (EASD); European Association for the Study of Obesity (EASO). EASL-EASD-EASO clinical practice guidelines on the management of metabolic dysfunction-associated steatotic liver disease (MASLD). J Hepatol 2024;81:492–542. 38851997 10.1016/j.jhep.2024.04.031

[27] Ioannidou A Alatar S Schipper R. Hypertrophied human adipocyte spheroids as in vitro model of weight gain and adipose tissue dysfunction. J Physiol 2022;600:869–83. 34387376 10.1113/JP281445

[28] Murata T Yamaguchi M Kohno S. Regucalcin enhances adipocyte differentiation and attenuates inflammation in 3T3-L1 cells. FEBS Open Bio 2020;10:1967–84. 10.1002/2211-5463.12947 PMC7530391 32783343

[29] Abulikemu A Zhao X Xu H. Silica nanoparticles aggravated the metabolic associated fatty liver disease through disturbed amino acid and lipid metabolisms-mediated oxidative stress. Redox Biol 2023;59:102569. 36512914 10.1016/j.redox.2022.102569 PMC9763688

[30] Drori A Rotnemer-Golinkin D Avni S. Attenuating the rate of total body fat accumulation and alleviating liver damage by oral administration of vitamin D-enriched edible mushrooms in a diet-induced obesity murine model is mediated by an anti-inflammatory paradigm shift. BMC Gastroenterol 2017;17:130. 29179679 10.1186/s12876-017-0688-4 PMC5704499

[31] Jahn D Dorbath D Kircher S. Beneficial effects of Vitamin D treatment in an obese mouse model of non-alcoholic steatohepatitis. Nutrients 2019;11:77. 30609782 10.3390/nu11010077 PMC6356425

[32] Patel YA Henao R Moylan CA. Vitamin D is not associated with severity in NAFLD: results of a paired clinical and gene expression profile analysis. Am J Gastroenterol 2016;111:1591–98. 27644736 10.1038/ajg.2016.406 PMC5331905

[33] Shojaei Zarghani S Soraya H Alizadeh M. Calcium and vitamin D3 combinations improve fatty liver disease through AMPK-independent mechanisms. Eur J Nutr 2018;57:731–40. 27988847 10.1007/s00394-016-1360-4

[34] Grinberg L Dabbah Assadi F Baum G. Beneficial effect of Vitamin D on Non-Alcoholic Fatty Liver Disease (NAFLD) progression in the zebrafish model. Nutrients 2023;15:1362. 36986092 10.3390/nu15061362 PMC10052639

[35] Reda D Elshopakey GE Albukhari TA. Vitamin D3 alleviates nonalcoholic fatty liver disease in rats by inhibiting hepatic oxidative stress and inflammation via the SREBP-1-c/PPARα-NF-κB/IR-S2 signaling pathway. Front Pharmacol 2023;14:1164512. 37261280 10.3389/fphar.2023.1164512 PMC10228732

[36] Powell EE Wong VW Rinella M. Non-alcoholic fatty liver disease. Lancet 2021;397:2212–24. 33894145 10.1016/S0140-6736(20)32511-3

[37] Foerster F Gairing SJ Müller L. NAFLD-driven HCC: safety and efficacy of current and emerging treatment options. J Hepatol 2022;76:446–57. 34555422 10.1016/j.jhep.2021.09.007

[38] Kim TH Yun SG Choi J. Differential impact of serum 25-Hydroxyvitamin D3 levels on the prognosis of patients with liver cirrhosis according to MELD and child-pugh scores. J Korean Med Sci 2020;35:e129. 32419396 10.3346/jkms.2020.35.e129 PMC7234861

[39] Kempinska-Podhorodecka A Adamowicz M Chmielarz M. Vitamin-D receptor-gene polymorphisms affect quality of life in patients with autoimmune liver diseases. Nutrients 2020;12:2244. 32727130 10.3390/nu12082244 PMC7469002

